# Clinical trials in allergen immunotherapy in the age group of children and adolescents: current concepts and future needs

**DOI:** 10.1186/s13601-020-00314-1

**Published:** 2020-04-24

**Authors:** O. Pfaar, R. Gerth van Wijk, L. Klimek, J. Bousquet, P. S. Creticos

**Affiliations:** 1 Department of Otorhinolaryngology, Head and Neck Surgery, Section of Rhinology and Allergy, University Hospital Marburg, Philipps-Universität Marburg, Marburg, Germany; 2 Section of Allergology, Department of Internal Medicine, Erasmus Medical Center, Rotterdam, The Netherlands; 3 Center for Rhinology and Allergology, Wiesbaden, Germany; 4 MACVIA-France, Contre les Maladies Chroniques pour un Vieillissement Actif en France, European Innovation Partnership on Active and Healthy Ageing Reference Site, Montpellier, France; 5 INSERM U 1168, VIMA: Ageing and Chronic Diseases Epidemiological and Public Health Approaches, Villejuif, France, Universite Versailles St-Quentin-en-Yvelines, UMR-S 1168, Montigny le Bretonneux, France; 6 Division of Allergy & Clinical Immunology, Johns Hopkins University School of Medicine, Baltimore, MD 21224 USA; 7 Creticos Research Group, Crownsville, MD 21032 USA

**Keywords:** Allergic rhinitis, Allergen immunotherapy, Clinical trials, Endpoints, Placebo, Children, Adolescents

## Abstract

Allergen immunotherapy (AIT) is the only treatment option available for allergic patients with disease-modifying intention. Both efficacy and safety has been demonstrated for multiple trials in children, adolescents and adults. Though regulatory requirements for marketing authorization have been clearly outlined and an increasing number of high quality trials has been initiated, multiple concepts and details in study design may be further elaborated, harmonized and improved. An international group of experts in the field of AIT has thoroughly reviewed and discussed current concepts and provided an outlook on further improvement especially in the age group of children and adolescents. Emphasis of the group’s discussion as a basis for this article was put on (i) the regulatory background of marketing authorization of AIT products including the ‘Pediatric Investigational Plan’, (ii) patient reported outcomes and endpoints in AIT trials, (iii) considerations regarding the ‘minimal clinically important difference’, (iv) the role of placebo effects in AIT clinical trials and clinical routine and (v) the potential of mobile Health for future development of AIT. Current concepts in AIT have been optimized throughout the recent decades, but there remains room for improvement e.g., in the topics outlined in this article.

## Introduction

Throughout the recent 100 years after the inauguration of Allergen immunotherapy (AIT) [1] as the only treatment option available modulating the underlying immunological cause of the disease [2] and the first double-blind placebo-controlled trial on AIT for grass pollen allergic patients performed in 1954 by Frankland et al. [3], a broad body of evidence for its efficacy and safety has been found subsequently in both pediatric and adult allergic patients [4–8]. An increasing number of clinical trials has been performed following the regulatory prerequisites for marketing authorisation (MA) by authorities such as the European Medicines Agency (EMA) or the Center for Biologics Evaluation & Research (CBER) of the Food and Drug Administration (FDA) in the US [9]. Principles for study-design and evaluation of results have been outlined by authorities such as the “*Guideline on the Clinical Development of Products for Specific Immunotherapy for The Treatment of Allergic Diseases* (CHMP/EWP/18504/2006)” of the EMA [10]. However, several methodological details have not been specified in these regulatory guidance and much emphasis has been put in the standardisation and improvement of trial design by the European Academy of Allergy and Clinical Immunology (EAACI) (summarized in [11]). Moreover, still there is some room for improvement in general and in particular in clinical trials in the pediatric population [12]. Therefore, the following aims to outline current challenges in trial design in AIT in general with a special focus on the needs and specifications in the pediatric age with Table 1 summarizing the current status and future needs of the examples of current concepts. As such, the review highlights recent discussion of a panel of experts in AIT and updates a series of scientific discussion [12].

**Table 1:** Current status and future needs of current concepts in AIT

Methodological topic addressed in this review	Current status	Future needs
The regulation of AIT products and Pediatric Investigational Plan	AIT products are regulated by the European Medicines Agency (EMA) in Europe and the Food and Drug Administration (FDA) in the US following similar principles regarding the clinical documentation in principle. Marketing authorization for AIT products in children have to be in line with the “Pediatric Investigational Plan” of the EMA	Solutions regarding the feasibility of clinical trials in children should be elaborated by all relevant stakeholders in the field of AIT
Patient reported outcomes and clinical endpoints in AIT trials	Patient Reported Outcomes and clinical endpoints in AIT trials should be harmonized and further evaluated re. their composition and clinical relevance. Though academy has proposed further recommendations for adult patients, definition of ideal endpoints in the pediatric population is still lacking	Further efforts should be put into the development and standardization of clinical endpoints suitable for children in pediatric AIT trials. Symptom-medication scores need to be defined with a specific emphasis on pre-school children
Minimal clinically important difference in AIT trials and clinical relevance	The aim of AIT trials is to demonstrate clinical efficacy and safety of investigated products. However, the minimal magnitude of the treatment effect size which is clinically relevant to the patient (minimal clinically important difference (MCID)) is not thoroughly validated and therefore justified for almost all endpoints in AIT clinical trials	There is a clear need for further exploration of MCID in AIT trials for multiple endpoints and this is in particular important in pediatric AIT trials to improve quality and feasibility of trials in this age group
Placebo effects in AIT trials and considerations for trial design improvement	Placebo mechanisms play an inevitable role in the field of AIT. These effects should be used within the whole treatment concept in clinical routine on one hand, but may also have some bias on conclusions drawn from clinical trials in AIT where they should be reduced to the most minimal level	Multiple effects of “the” placebo mechanisms in AIT treatment should be better evaluated and understood. These effects may play an important role especially in AIT of pediatric patients
mHealth in the AIT precision medicine approach	mHealth continuously plays an important role in daily healthcare delivery in general. Mobile used Apps may become a very effective tool in clinical trials in AIT (e.g., selection of patients, readouts of efficacy measures and others) and also for monitoring patients in daily life routine	While Apps and other of mHealth tools are steadily improving (‘machine learning’), easy and smart applications especially for monitoring of children and adolescents are needed. Even more this technique would allow a more precise evaluation of any kind of medical intervention such as e.g., AIT in pediatrics

## The regulation of AIT products and Pediatric Investigational Plan

For MA, AIT products need to be approved by national competent authorities in different regions of the world including the European Union (EU) and the United States of America (USA) in general [9, 13]. While there is a wide heterogeneity in the demands for regulatory approval of AIT products—both within the EU and between EU and USA—the requirements for high quality clinical data for granting market access are a common feature and follow the guidelines on Good Clinical Practice (GCP) in the conduct of clinical trials [9, 14].

In the EU, the Clinical Trials Directive [15] implemented GCP as a mandatory requirement for the conduct of state-of-the-art randomized, double-blind placebo-controlled (DBPC-) clinical trials and to document them in an European database, similar to what is required in the US [9, 14]. Approval of AIT products involves the independent assessment as to whether or not a specific product shows a favorable risk–benefit profile as the EU defines AIT products as medicinal products according to Directive 2001/83/EC [15], and with some exemptions AIT products need MA. A unique combination of national regulatory competent authorities in the EU work together in a network to regulate market access of medicinal products with the EMA, that is responsible for the coordination of the different MA procedures including the centralized procedure. To date, most AIT products use national MAs. Extending a national marketing authorization to additional member states can be organized via the Mutual Recognition Procedure (MRP), in which the prior country acts as Reference Member State (RMS) and will provide assessment reports to subsequent countries (Concerned Member States, CMS). The Decentralized Procedure (DCP) is now the preferred route for achieving MAs in multiple EU member states, as it allows faster decision and potential approval, as there is no requirement for a national authorization to precede the DCP [9, 14].

In the United States, the CBER of the FDA grants MAs of AIT products. To date, unmodified standardized and non-standardized aqueous aeroallergen extracts have a MA with the majority for subcutaneous AIT products [16]. In addition, sublingual tablets have also gained MA in the US [17]. To date, no formal guidance on clinical development demands on AIT products in the US has been proposed by the US CBER with the consequence that each product’s development is assessed and regulated individually [16]. Recent MA of sublingual tablets has, however, followed similar prerequisites regarding the clinical documentation as the guidance of the EMA.

Following the ‘*Pediatric Investigational Plan*’ (PIP), the Pediatric Committee (PDCO) of the EMA requests evidence for clinical efficacy from at least one longterm (3-year double-blind, placebo-controlled and 2-year blinded follow-up) study for an AIT product of the company’s portfolio [13, 18]. However, practical and ethical concerns arise from this regulatory demand as it has been shown extremely difficult for clinical trial centers to enroll a sufficient number of children for these trials [19], and long-term efficacy has been demonstrated for only a few products in this age group which would not be provided to children participating in such a trial [13, 20]. Therefore, there is an urgent need for further development of alternatives in AIT trial design in children and solutions should be made by all stakeholders involved such as pediatricians and allergists, regulatory authorities and manufacturers of AIT products [12, 13, 20].

In conclusion, regulatory prerequisites for recent MA have been comparable between Europe and the US and in principle are based on evidence for clinical efficacy and safety demonstrated in clinical trials following modern study-designs. In Europe, however, MA in the pediatric population will have to follow the ‘*Pediatric Investigational Plan*’ which raises concerns about the feasibility of these trials and further discussion.

## Patient reported outcomes and clinical endpoints in AIT trials

Although many studies have included school children and adolescents in their pivotal trials, there are very few pediatric studies [6]. However, children may react differently from adults to AIT and there are no specific patient reported outcomes (PROs) for children. These considerations are particularly important for pre-school children.

Clinical trials include primary outcome measures that provide the most clinically relevant and convincing evidence [15]. As the methodology of choosing and assessing outcome measures in AIT studies varies widely, an EAACI taskforce collected all available clinical measures of efficacy and made recommendations for their use in AIT trials [21]. The task force recommended a combined symptom and medication score as a harmonized and clinically justified primary outcome that has been followed in an increasing number of recent AIT trials [22, 23]. Notably, this combined proposed score better discriminates between active and placebo treatment than the evaluation of symptom and medication scores separately [24]. This score needs to be tested and compared with other scores. Mobile Health (mHealth-) tools may be appropriate to validate such scores.

However, the expert panel of the EAACI clearly highlighted the unmet need of further validation of his proposed endpoint especially in children where symptom and medication reporting is probably not the best methodological option to detect clinical efficacy of interventions as reporting is often given by parents [21], and further disadvantages of this approach can be argued for this age group, e.g., the fact that the amount of the concomitant rescue medication taken may not directly represent the level of the child’s symptom severity [12].

Secondary endpoints in AIT trials comprise other PROs e.g., subjective outcome measures such as individual symptom or medication scores, visual analogue scales (VAS), health related quality of life (HRQL) assessment, well days and severe days, global assessment, patient satisfaction and measures of rhinitis control [21, 25]. Some of those are even validated for pediatric and adolescent patients as the Rhinoconjunctivitis Quality of Life Questionnaire (RQLQ) [26, 27]. Besides, the VAS score has been validated for adults [28], but not for children although the VAS is an instrument that could be easily implemented in pediatric trials and reporting would be facilitated in children as reported in one clinical trial [29].

Objective secondary endpoints also comprise assessment by nasal or conjunctival provocation or by exposure in allergen exposure chambers (AEC), extrapolating clinical effects of interventions to natural situation under natural allergen exposure, e.g., in the season [30–32]. Especially, trials in the latter would have a potential to reduce the high number of pediatric patients needed to fulfil regulatory requirements for marketing authorization as demanded by the PDCO as outlined above [18], but apparently further technical and clinical validation in both pediatric and adult population is strictly needed before they will be accepted as pivotal for MA [10, 32].

In addition, measurement of specific IgG4 or blocking antibody activity are commonly used as secondary parameters in clinical trials in AIT. However, only the increase in blocking antibody activity has been associated with symptom improvement and further research of potential biomarkers in AIT has been highlighted as an important unmet need by the EAACI [33].

In conclusion, in spite of a consented position from academy for the preferred primary endpoint in AIT trials there remains a need to identify the best compositions of outcome measures in adult and pediatric AIT phase III trials. Ideally, such panels of endpoints may be limited to the CSMS as proposed by the EAACI in the combination with perhaps two confirming secondary measures besides the global evaluation of safety. However, clinical measures in pediatric trials may differ from adult study designs and an international harmonized standard especially for trials in this age population is warranted and should be elaborated by clinicians, regulatory and ethical authorities.

## Minimal clinically important difference in AIT trials and clinical relevance

In line with regulatory prerequisites as the “*Guideline on the Clinical Development of Products for Specific Immunotherapy for The Treatment of Allergic Diseases*” of the EMA efficacy of AIT products has to be demonstrated in large clinical (so called pivotal) trials [10]. Because of the size of these studies, there is an obvious risk that small but statistically significant inpatient changes or between‐patient differences may be observed. The question, however, remains as to whether such observations are relevant for patients and clinicians.

Calculating the minimal clinically important difference (MCID) could be a solution to overcome this requirement. The MCID has been defined as the smallest difference in the scores of a measure that is perceived by patient to be beneficial or harmful [21]. The MCID has been established mostly for a few secondary outcome measures such as the RQLQ (0.5) [34], the mini RQLQ (0.7) [35], the VAS [28] and the CARAT score for adults [36] and children [37]. In one AIT study the MCID of symptom scores has been calculated as 1.1–1.3 (rounded to 1) [38]. However, one has to bear in mind that the MCID represents a clinically relevant *change* in an outcome measure, which implies a comparison between a baseline measurement and a measurement after 1 or more years of treatment. For instance, while a change in the RQLQ of 0.5 on a 7 points scale is clinically relevant, a difference of 0.5 between the active or placebo group does not constitute a similar meaning [21].

The inclusion of MCIDs in clinical trials has several advantages [39]. Not only is the patient’s perspective taken into account, clinicians are more able to interpret the results of a trial and to understand the success or the lack of success of an intervention. MCIDs help to estimate the sample size and it is also easy to calculate the proportion of patients who achieve the MCID. From this proportion, the number needed to treat can be derived. Therefore, accepted ways to define and include clinical relevance will enhance the quality of clinical trials and contribute to a better positioning of new interventions into clinical practice. This is of utmost importance especially in clinical trials of AIT in the pediatric population with the aim to minimize the number of enrolled subjects due to ethical reasons.

This MCID described above has to be distinguished from the desired difference between placebo and active treatment of ≥ 20% stated by the World Allergy Organization (WAO) [40] and by the “Global Allergy and Asthma European network” (GA2LEN) of the initiative “Allergy and its Impact on Asthma” (ARIA) [41]. However, this clinical minimal difference has not been determined with sufficient precision.

In contrast, for MA, the suggested guidance of the CBER branch of the FDA outlines two criteria that, in tandem, need to be met by a company submitting a Biological License Application (BLA) for a product to receive FDA regulatory approval. These include a desired difference between placebo and active treatment of ≥ 15% in the Total Combined Score (TCS) and evidence for a MCID that is further supported by clear separation of the active drug vs placebo group based on the 95% Upper Bound CI (≤ 10%) [42, 43].

In conclusion, MCID in clinical trials allows a sufficient orientation of a medical intervention such as e.g. AIT regarding the clinical (relevant) efficacy. However, a thorough validation of the MCID for different clinical endpoints proposed is needed. This is in particular the case for AIT trials in children and adolescents in which a clinically justified MCID will improve the quality (and the feasibility) of the trial. As MCIDs are used in RCTs that may not reflect real life, observational studies should complement RCTs for real world evidence.

## Placebo effects in AIT trials and considerations for trial design improvement

There is no generally accepted definition of when a treatment-intended substance is a placebo nor what is a placebo effect. In general, the definition by Brody and Moerman is widely used: “*The placebo effect is most usefully defined as a positive healing effect resulting from the use of any healing intervention presumed to be mediated by the symbolic effect of the intervention for the patient.*” [44]. However for use in clinical trials, the definition by Gøtzsche may be more useful: “*The placebo effect is the difference in outcome between a placebo treated group and an untreated control group in an unbiased experiment.*” [45]. By that mean, in scientific studies placebo is considered to be a substance without pharmacological effects itself which is provided to a control arm of patients. This aims to reduce any kind of unspecific psychological and physical benefit an individual may gain through treatment which will bias the outcome of a trial [12]. In line with current regulatory demands, double-blind placebo-controlled (DBPC) clinical trials remain the gold standard for quantifying the efficacy of medical interventions [10, 46].

This is in particular the case in AIT clinical trials to ensure an unbiased reporting on its efficacy and tolerability by blinding of possible stratification of patients, reporting of outcomes as well as analysis and interpretation of data [10, 46]. Clinical endpoints, e.g., the CSMS of the EAACI [21] are predominantly subjective which influences the robustness of these trials negatively. They are dependent on the subject and the investigator applying their judgment to score and grade their internal (subjective) feelings. Objectively measurable endpoints like laboratory results are less affected by both the investigator and the patient.

There is good evidence that placebo effects may be more marked in children than in adults although fewer placebo-controlled trials are conducted in children in general [47]. However, in the field of AIT there are no trials published in the pediatric population with a baseline period (without intervention before randomization [48]) analyzed which would be helpful to indicate the impact and extent of placebo-effects in this age group.

In contrast to clinical trials with AIT in which results can be skewed due to the often pronounced placebo effect, these poorly understood, potentially neuro-immunological, mechanisms [12] are highly effective and desired in the whole treatment concept of allergic patients in daily clinical routine in general.

In conclusion, placebo mechanisms play an inevitable role in medicine in general and in particular in the field of AIT. These effects have an important impact on the treatment effect in AIT which is desirable in the clinical routine, but also dilutes signals from clinical trials. Therefore, further investigations and positions from academia such as a Task Force of the EAACI are of utmost importance.

## mHealth in the AIT precision medicine approach

mHealth (mobile health) refers to the use of mobile and wireless devices to support healthcare delivery and improve health outcomes. mHealth has evolved from eHealth, the use of information and communication technology (ICT) for health services and information transfer. According to the World Health Organization (WHO) [49], mHealth can transform the face of health service delivery and may play a central role in future evolution of “*precision medicine*” [50]. The rapid advances in mobile technologies and applications lead to a rise in new opportunities for the integration of mobile health into health and care. Many of these tools can be applied to the study and management of allergies. A recent review has provided examples of Apps used in allergic rhinitis [51]. These tools can be used for AIT in the selection and stratification of patients, the assessment of early benefits, evaluation of efficacy during the course and at the end of AIT, and in the follow-up after AIT has been terminated [52]. However, current mHealth tools cannot be used in young children as there are some ethical issues below 15 to 18 years depending on the countries and further development of smart devices in this age population would be preferable.

In line with current international guidelines in AIT, patients should be selected on the basis of the severity of the disease and on further prerequisites, e.g., a good treatment adherence [53–55]. This can easily be achieved using electronic diaries obtained by cell phones as demonstrated in MASK-air^®^—a free online app developed for IOS and Andoid-systems by MACVIA-ARIA, an international sentinel network for allergic rhinitis [56–58]. Such diaries should include the full list of medications. After a single year of survey, physicians can assess whether severe uncontrolled upper airway disease (SCUAD) [53] is present and could initiate AIT if (i) symptoms are associated with allergy season, (ii) adherence to pharmacologic treatment is achieved, (iii) the duration of uncontrolled symptoms was long enough and (iv) an impact on work or school productivity was observed. Moreover, asthma and eye symptoms can be recorded, as in MASK-air^®^ [57] and other Apps allowing to evaluate the role of multi-morbidity [59]. The same approach can be proposed for the follow up of patients on AIT to assess its efficacy as suggested by a panel of international experts in an AIT Position Paper [21, 52].

An electronic clinical decision support system (CDSS) is a health information technology (IT) system designed to assist clinicians and other health care professionals in clinical decision-making. In medicine, CDSSs have become a major topic in artificial intelligence. The AR algorithm has been digitalized in tablets for health care professionals [60]. A CDSS can reduce the burden that exponentially-expanding clinical knowledge and care complexity places on clinicians, other health care professionals or patients [61]. Embedding the results of Apps in and e-CDSS will help physicians to decide when to start and stop AIT.

In conclusion, the use of mobile and wireless devices is increasingly enhancing healthcare delivery and improving health outcomes (mHealth). Apps may be used for optimization of AIT by the selection and stratification of patients, the assessment of onset of action, evaluation of efficacy during the treatment course and after cessation. As such this technology will enable a better documentation of treatment effects in pediatric trials assumed that smart technical solutions can be further developed for this age group.

## Conclusion

Regulatory authorities request a clinical documentation of efficacy and safety for marketing authorization following guidance such as e.g. the “*Guideline on the Clinical Development of Products for Specific Immunotherapy for The Treatment of Allergic Diseases* (CHMP/EWP/18504/2006)” of the EMA. However, some of methodological common standards in AIT clinical trial design need further exploration and thorough consideration. This review reports scientific discussion held by German, French, Dutch and US American experts in the field of AIT regarding improvement of concepts in AIT trial design. Emphasis is put on the regulatory situation with a special focus on the “*Pediatric Investigational Plan*” for AIT, on patient reported outcomes and clinical endpoints in both adult and pediatric trials, on the importance of further investigation of the “*minimal clinically important difference*” in the evaluation of clinical efficacy as demonstrated in AIT trials, on the role of the placebo effect as one component of the whole treatment effect, and the potential of mHealth for further elaboration of the AIT benefit in the era of precision medicine. Pediatric specifications are emphasized for all of these concepts. Future work may also focus on additional key research areas in this particular age-group such as e.g., trial designs for prevention of allergies, polysensitized children/adolescents, asthma endpoints etc. A better understanding and improvement of these concepts are key in aiming to confirm AIT as the only-disease modifying treatment option available especially in children.

## Data Availability

Not applicable.

## Abbreviations

AEC: Allergen exposure chambers
AIT: Allergen immunotherapy
ARIA: Allergy and its Impact on Asthma
CBER: Center for Biologics Evaluation & Research
CDSS: Clinical decision support system
CMS: Concerned Member States
CSMS: Combined symptom and medication score
DCP: Decentralized Procedure
EAACI: European Academy of Allergy and Clinical Immunology
EMA: European Medicines Agency
EU: European Union
FDA: Food and Drug Administration
GA2LEN: Global Allergy and Asthma European network
GCP: Good Clinical Practice
HRQL: Health related quality of life
ICT: Information and communication technology
IT: Information technology
MA: Marketing authorisation
MCID: Minimal clinically important difference
mHealth: Mobile Health
MRP: Mutual Recognition Procedure
PDCO: Pediatric Committee
PIP: Pediatric Investigational Plan
PRO: Patient reported outcomes
RMS: Reference Member State
RQLQ: Rhinoconjunctivitis Quality of Life Questionnaire
SCUAD: Severe uncontrolled upper airway disease
USA: United States of America
VAS: Visual analogue scales
WAO: World Allergy Organization
